# A Screening and Referral Intervention for Food Insecurity Among Older Emergency Department Patients

**DOI:** 10.1093/geroni/igaa057.121

**Published:** 2020-12-16

**Authors:** Aileen Aylward, Timothy Platts-Mills, Liane Wardlow, Conor Sullivan, Jessa Engelberg Anderson, Andrea Morris, Rayad Shams

**Affiliations:** 1 University of North Carolina at Chapel Hill, Chapel Hill, North Carolina, United States; 2 West Health Institute, La Jolla, California, United States; 3 ServiceNow, San Diego, California, United States; 4 West Health Institute, San Diego, California, United States

## Abstract

Food insecurity is prevalent among older adults, negatively impacts health, and may increase healthcare utilization. Risk factors include poverty, lack of transportation, and social isolation. Community-based services may mitigate food insecurity and other social risk factors. However, identifying those at risk and connecting them to services can be challenging. We implemented a screening and referral program in an Emergency Department (ED) to identify older adults facing food insecurity and connect them to a local Area Agency on Aging (AAA), which arranged and tracked delivery of community-based services. ED nursing assistants used the Hunger Vital Sign screener to assess food insecurity in patients aged 60 years and older. ED Care Managers (CMs) saw all who screened positive and made referrals to the AAA. The AAA conducted an intake assessment and arranged services. Patients were contacted three months after their ED visit to evaluate health, quality of life, and satisfaction with services. Of 423 patients screened over 7 months, 45 (11%) reported food insecurity. Of those, 25 were referred to the AAA. Patients were not referred to the AAA due to CM inability to make a referral (7), declining services (4), or other reasons (11). The AAA reached 21 patients and 9 received at least one service. Of those, 5 were reached for follow-up and reported satisfaction with services. The most frequently requested service was Meals on Wheels (10). Food insecurity is common among older ED patients. An ED-AAA partnership is feasible and connects older adults to beneficial services in their communities.

